# Bridging the gap between gastric pouch and jejunum: a bariatric nightmare

**DOI:** 10.1186/s12893-015-0043-z

**Published:** 2015-05-30

**Authors:** Noëlle Geubbels, Ingrid Kappers, Arnold W. J. M. van de Laar

**Affiliations:** Department of Surgery, Slotervaart hospital, Amsterdam, The Netherlands

**Keywords:** Laparoscopic Roux-en-Y gastric bypass surgery, Short mesentery, Retrocolic/retrogastric route, Intraoperative event

## Abstract

**Background:**

Even in a large volume bariatric centre, bariatric surgeons are sometimes confronted with intraoperative anatomical challenges which force even the most experienced surgeon into a pioneering position. In this video we present how a large gap of approximately 8 cm is bridged by applying several techniques that are not part of our standardized surgical procedure.

**Case presentation:**

After creation of a 20 mL gastric pouch we discovered that the alimentary limb could not be advanced further cranially due to a very short a thick jejunal mesentery in a 49 year old male patient during laparoscopic Roux-en-Y gastric bypass (LRYGB) surgery. By dissecting the gastro-oesophageal junction form the crus, stretching the gastric pouch, transecting the jejunal mesentery, using a retrocolic/retrogastric route, and creating a fully hand-sewn gastrojejunostomy we were able to safely complete the LRYGB. Drains were left near the gastrojejunostomy and the patient was kept nil by mouth for 5 days. On the 5th postoperative day radiographic swallow series were obtained which revealed no sign of leakage. The patient was discharged in good clinical condition on the 6th postoperative day. To date, no complications have occurred. Weight loss results are −31.5 % of the preoperative total body weight.

**Conclusions:**

When confronted with a large distance between the gastric pouch and the alimentary limb, several techniques presented in this video may be of aid to the bariatric surgeon. We stress that only experienced bariatric surgeon should embark on these techniques. Inspecting the alimentary limb before the creation of the gastric pouch may prevent the need for such complex techniques.

**Electronic supplementary material:**

The online version of this article (doi:10.1186/s12893-015-0043-z) contains supplementary material, which is available to authorized users.

## Background

The Slotervaart Hospital is a teaching hospital in Amsterdam, The Netherlands. We commenced our bariatric program in 2007, gradually expanding in surgical volume about 900 patients annually in 2014. Over the course of these years, many modifications were made to the bariatric program in order to facilitate this expanding number of patients. We have changed our surgical technique, implemented an enhanced recovery, or ‘fast track’ program and trained 2 new residents in to bariatric surgeons, all leading to improvements in patient safety and flatter surgical learning curves [1, 2]. Still, experience and standardization does not rule out that one is sometimes confronted with an exceptional surgical situation. The aim of this video is to demonstrate how a large distance between the alimentary limb and the gastric pouch can be overcome with the aid of several surgical techniques.

## Case presentation

### Brief description of our standardized surgical technique

A 20 mL gastric pouch is created with the use of two to three 60 mm linear staplers (Endo GIA, Covidien and Dublin, Ireland). At approximately 40 cm proximal to the ligament of Treitz the jejunum is grasped and mobilized to the gastric pouch. The posterior side of the gastrojejunostomy is stapled with a 30 mm linear stapler and the anterior side is hand sewn with an absorbable unidirectional barded 3—0 V-Loc™ suture (Covidien, Dublin, Ireland). At about 150 cm a fully stapled jejunojejunostomy is created with two linear 60 mm staplers. Then the jejunum is transected between the two anastomoses using a 60 mm linear stapler without division of the mesentery. The gastrojejunal anastomosis is tested for leakage with methylene blue through the orogastric tube. There is no routine placement of drains. The orogastric tube is removed at the end of surgery. The patients are allowed a clear fluid diet when fully recovered from anaesthesia. No routine radiographic swallow series are obtained. All patients receive subcutaneous low molecular weight heparin during the first two weeks after surgery as thromboprophylaxis. The patient’s diet is gradually expanded to a full liquid during their admission and continued for two weeks. All patients receive supplementary vitamins and a proton pump inhibitor.

#### The patient

In December 2012, a 49 year old male was scheduled for laparoscopic Roux-en-Y gastric bypass surgery (LRYGB). At the time of surgery his weight was 138.2 kg with a Body Mass Index (BMI) of 45.1 kg/m^2^. His past medical history revealed Obstructive Sleep Apnea (OSA), for which he uses Continuous Positive Airway Pressure (CPAP) therapy, Chronic Obstructive Pulmonary Disease (COPD) stage GOLD 2, Post-Traumatic Stress Disorder (PTSD) after a car accident and non-ST elevated myocardial infarction (NSTEMI) for which he underwent a successful percutaneous coronary intervention (PCI) of the ramus circumflexus of his left coronary artery. During his medical screening the patient was diagnosed with Type 2 Diabetes Mellitus (T2DM) ‘de novo’, which was treated with oral medication. The patient’s cardiac, respiratory, and endocrinological function were well assessed prior to surgery and optimally regulated.

During the surgery of this patient we where forced to make several deviations from our standardized protocol. The subheadings correspond to the headings of the accompanying video (Additional file 1).

#### Identification of the ligament of Treitz and discovery of the short mesentery

After positioning the patient, the introduction of the ports, the creation of a 20 ml gastric pouch and the division of the (very bulky) omentum, the ligament of Treitz is identified. When measuring the jejunum from the ligament of Treitz is becomes apparent that the mesentery is very short. It is not possible to mobilize the jejunum over the transverse colon (antecolic route) and the remnant stomach (antegastric route). The distance between the jejunum is about 8 cm. We measured this distance with the aid of marking on our graspers.

#### Transection of the jejunum and division of the mesentery

In order to create the alimentary limb, the jejunum is transected at the point where the distance to the proximal gastric pouch is the shortest. To further mobilize the alimentary limb, the mesentery is divided with the ultracision harmonic scalpel.

#### Placement of a marker stitch in the alimentary limb

A marker stitch (vicryl 2.0, Ethicon Inc. Johnson, & Johnson, New Brunswick, New Jersey, USA) is placed to mark the alimentary limb. Later, this stitch will be used to pull the limb through the retrocolic route.

#### Dissection of the gastro-oesophageal junction from the crus

To lengthen the proximal gastric pouch, first the gastro-oesophageal junction is dissected from the crus by transecting the phrenoesophageal ligament on both sides. This technique lengthens the proximal pouch about 2 cm. Because of the traction caused by the gastrojejunostomy, we decided no fixation was needed.

#### Stretching the pouch

Consecutively the pouch is stretched. This is achieved by grasping the pouch on both sides and to pull the pouch caudally for about 1 min. This manipulation of the pouch will gain another 0.5 cm.

#### Creation of the retrocolic route through the mesocolon

The retrocolic route is created starting on the caudal side of the mesocolon using the ultracision harmonic scalpel.

#### Pulling the alimentary limb through the retrocolic/retrogastric route

When completed, the marker stitch is placed in the retrocolic ‘tunnel’. The mesocolon is folded down. Cranially of the mesocolon, the marker stitch is found. The jejunum is retracted by pulling the marker stitch whilst retracting the gastric remnant caudally.

#### Creation of the hand—sewn gastrojejunostomy – The posterior sutures

It becomes apparent that a stapled anastomosis between the gastric pouch and the jejunum is not preferable due to the foreseen tension on this stapler line. Therefore, we decided to make a full hand—sewn anastomosis with V-Loc™ sutures.

#### Creation of the gastro—and the jejunotomy

A defect is created in the gastric pouch and the jejunum using the ultracision harmonic scalpel.

#### Introduction of the 34 Ch orogastric tube

A 34 Ch orogastric tube is passed through the gastric pouch and into the alimentary limb of the jejunum.

#### Creation of the anterior hand sewn anastomosis

A running V-Loc™ suture is used to close the anterior part over the tube in order to ensure the patency of the anastomosis.

####  Leak test: leak at the right lateral side of the anastomosis

The first leak test with methylene blue through the orogastric tube shows a leak on the right lateral side of the anastomosis.

####  Oversewing the right lateral side of the anastomosis

The defect is over sewn with the remaining V-Loc™ sutures.

#### Final leak test: no leakage

The final leak test revealed no leakage.

The creation of the jejuno—jejunostomy went according to our standardized protocol. The mesenteric, mesocolic, and Petersen’s defect were closed using the hernia stapler. A 27 Fr drain was left lateral to the gastrojejunostomy. The patient was kept nil by mouth for 5 days and fed parenteral.

## Results

During the whole admission, the patient was in a good clinical condition: he was hemodynamically stable and showed no signs of anastomotic leakage. On the 5th postoperative day radiographic swallow series where obtained, which showed no signs of leakage. The drain was unproductive during the whole admission. After the swallow studies the drain was removed. On the 6th postoperative day, the patient was discharged with a full liquid diet. To date, no signs of any complication have emerged (we were especially watchful for signs of stricture, stenosis, and internal herniation). Weight loss results are good: 31.5 % of total preoperative body weight after 12 months. Patients’ blood glucose values returned to normal with discontinuation of all anti-diabetic medications.

## Discussion

This video provides a step—to—step guidance on how to solve the rare, but technically demanding intraoperative complication of a large gap between the gastric pouch and the alimentary limb of the jejunum. By dissecting the gastro-oesophageal junction form the crus, stretching the pouch, dividing the mesentery of the jejunum, using the retrocolic/retrogastric route, and the creation of a total hand—sewn gastrojejunostomy were we were able to bridge the gap.

In stark contrast to the reports elaborating the benefits of LRYGB surgery, very little is known about the intraoperative complications. In large trials and reviews conversion rates up to 4.2 % percent are reported [3, 4], but number are seldom accompanied with a reason why the decision to conversion was made. This is strange considering the occurrence of ‘intraoperative events’ turns out to be an individual predictor for postoperative complications in a large study by Stenberg *et al.* [5]. This study also revealed that more than one third of the conversions were due to ‘difficult anatomic conditions’ [5]. The lack of reports on how to cope with intraoperative events force even the most experienced bariatric surgeons in to a pioneering position.

All techniques we describe are not part of our standardized surgical technique and may have disadvantages for the patient. Dissecting the gastro-oesophageal junction from the crus can cause a hiatal hernia, but since the traction in caudal direction from the gastrojejunostomy will prevent the pouch from moving cranially, we thing this is a minor concern. Any form of manipulation of the tissue of the pouch might cause bleeding, ischemia, or tearing. Therefore, stretching of the pouch is a subject of debate. We stress that only experienced surgeons can decide whether or not to apply this technique, based on ‘tissue feel’, and their ability to cope with the possible complications. Division of the mesentery, especially in a situation of increased tension, may result in bleeding, and consequent ischemia of the adjoining jejunum [6]. Furthermore, some authors stress that transecting the mesentery creates a large orifice and may become a potential hernia space [7], although this was not proven in a recent anatomical study [8]. In a survey executed amongst 215 American Society for Bariatric Surgery (ASBS) affiliated surgeons 64 % of the surveyed bariatric surgeons used the antecolic/antegastric route for the alimentary limb [9]. Eleven percent preferred the retrocolic/retrogastric route [9]. An advantage of the retrocolic/retrogastric route is that it is the shortest route for the alimentary limb to cranially reach the gastric pouch. A disadvantage is the need to create an extra opening in the mesocolon of the transverse colon to facilitate this route, hereby creating an extra orifice and potential hernia space [10]. Several studies report a decrease in internal hernia (IH) incidence when using the antecolic/antegastric route in comparison to the retrocolic route [11–14], although some authors report the lowest IH incidence using a retrocolic/retrogastric technique [15]. In this case we performed a hand—sewn gastrojejunostomy whilst our standardized technique is the linear stapling technique. We chose this technique over all others due to the decrease in surgical time compared to circular and hand—sewn anastomosis [16] and the high incidence of wound infections with circular stapling technique [17]. In this case, the traction on the anastomosis did not allow us to use linear stapling. In the ASBS survey 41 % of the surgeons indicated that they used the linear stapling technique to create the gastric pouch. In addition, 43 % used a circular stapler device and 21 % reported to make a hand sewn gastrojejunostomy [9]. Some studies found a higher rate of strictures with a hand sewn suturing technique in comparison to linear or circular stapling techniques [16], others found no difference [18].

Because of the increased tension on the gastrojejunostomy the risk of leak was high. As a safety measure, we left drains near the gastrojejunostomy, kept the patient nil by mouth and we obtained radiographic swallow series on the 5th postoperative day. It is doubtful if these precautions would have prevented a leak. The aim of these precautions was rather to decrease the severity in case of a leak and to detect a possible leak in an early phase.

From above it is clear that all applied techniques are inferior to our standardized technique and that they should only be used when confronted with an intraoperative event. Maybe even better is the prevention of such situations. This can be done by switching the order of the surgical steps. In our standardized technique the gastric pouch is created at the beginning of the procedure. Schauer *et al.* suggested the formation of the gastric pouch after the inspection and creation of the alimentary limb [17]. If we had adapted this technique, we could have created a longer pouch or –maybe even better—we could have converted to a sleeve gastectomy.

## Conclusion

This video report shows how a large distance between a newly created gastric pouch and the alimentary limb can be bridged, By dissecting the gastro-oesophageal junction from the crus, stretching the pouch, transecting the mesentery of the jejunum, using a retrocolic/ retrogastric route and creating a hand—sewn anastomosis we were able to bridge a 8 cm gap. All these manoeuvres are not part of our standard surgical technique as they are all associated with adverse patient outcome. We stress that only experienced bariatric surgeons should embark on these techniques. Inspection of the alimentary limb before pouch created might prevent the need for these complex techniques.

## Consent

Written informed consent was obtained from the patient for publication of this video and any accompanying moving or still images. A copy of the written consent is available for review by the Editor-in-Chief of this journal.

## Additional file

**Additional file 1:** Bridging the gap between gastric pouch and jejunum. (MOV 217 MB)
